# A novel role for RecA under non-stress: promotion of swarming motility in *Escherichia coli *K-12

**DOI:** 10.1186/1741-7007-5-14

**Published:** 2007-03-28

**Authors:** José-María Gómez-Gómez, Candela Manfredi, Juan-Carlos Alonso, Jesús Blázquez

**Affiliations:** 1 Departamento de Biotecnología Microbiana. Centro Nacional de Biotecnología, C/Darwin, 3, 28049-Madrid, Spain

## Abstract

**Background:**

Bacterial motility is a crucial factor in the colonization of natural environments. *Escherichia coli *has two flagella-driven motility types: swimming and swarming. Swimming motility consists of individual cell movement in liquid medium or soft semisolid agar, whereas swarming is a coordinated cellular behaviour leading to a collective movement on semisolid surfaces. It is known that swimming motility can be influenced by several types of environmental stress. In nature, environmentally induced DNA damage (e.g. UV irradiation) is one of the most common types of stress. One of the key proteins involved in the response to DNA damage is RecA, a multifunctional protein required for maintaining genome integrity and the generation of genetic variation.

**Results:**

The ability of *E. coli *cells to develop swarming migration on semisolid surfaces was suppressed in the absence of RecA. However, swimming motility was not affected. The swarming defect of a Δ*recA *strain was fully complemented by a plasmid-borne *recA *gene. Although the Δ*recA *cells grown on semisolidsurfaces exhibited flagellar production, they also presented impaired individual movement as well as a fully inactive collective swarming migration. Both the comparative analysis of gene expression profiles in wild-type and Δ*recA *cells grown on a semisolid surface and the motility of *lexA*1 [Ind-] mutant cells demonstrated that the RecA effect on swarming does not require induction of the SOS response. By using a RecA-GFP fusion protein we were able to segregate the effect of RecA on swarming from its other functions. This protein fusion failed to regulate the induction of the SOS response, the recombinational DNA repair of UV-treated cells and the genetic recombination, however, it was efficient in rescuing the swarming motility defect of the Δ*recA *mutant. The RecA-GFP protein retains a residual ssDNA-dependent ATPase activity but does not perform DNA strand exchange.

**Conclusion:**

The experimental evidence presented in this work supports a novel role for RecA: the promotion of swarming motility. The defective swarming migration of Δ*recA *cells does not appear to be associated with defective flagellar production; rather, it seems to be associated with an abnormal flagellar propulsion function. Our results strongly suggest that the RecA effect on swarming motility does not require an extensive canonical RecA nucleofilament formation. RecA is the first reported cellular factor specifically affecting swarming but not swimming motility in *E. coli*. The integration of two apparently disconnected biologically important processes, such as the maintenance of genome integrity and motility in a unique protein, may have important evolutive consequences.

## Background

Motility is an important bacterial niche colonization factor [1,2], thus many bacterial species are motile by flagellar rotation. In *Escherichia coli *flagella propel bacteria swimming in liquid medium or in semisolid agar media [3]. In response to chemotactic external signals, *E. coli *is able to direct its swimming movement towards a microenvironment that is optimal for its growth and survival [4]. When the conditions for swimming become adverse *E. coli *develops a different program for swarming motility [1,5-7]. In many bacterial species this flagella-driven coordinated motility is mediated by cell-cell communication mechanisms such as quorum sensing [8]. Thus, this bacterial migration activity is an intrinsically surface-linked phenomenon, leading to a change from an individual to a collective "social" behavior that allows the rapid exploration and colonization of surfaces [1,8,9].

Several environmental and nutritional factors promoting swarming behavior in some bacterial species such as *Proteus mirabilis *[10], *Serratia liquefaciens *[11], *Bacillus subtilis *[12], *Pseudomonas aeruginosa *[13,14] and *Salmonella enterica *Serovar Thyphimurium [15] have been described. In *E. coli*, however, the chemical and physical factors involved in swarming migration are still to be characterized. By contrast, although the relationship between several types of environmental stress and bacterial motility has been well studied in swimming [2,16-19], their effect on swarming motility has been poorly explored.

Bacteria need to cope with different types of stress in nature [20], environmentally induced DNA damage (e.g. UV irradiation) being a common one [21]. The relationship between DNA damage and colonial motility has not been previously addressed. RecA is a protein with a central role in DNA stability and repair under stress conditions leading to DNA damage [22-27]. RecA has multiple functions affecting different cellular processes, such as genetic recombination [22,28], the rescue of stalled or collapsed replication forks [25,29], and the replication of damaged DNA through translesion DNA synthesis (TLS) by DNA polymerase V (pol V) [30,31]. Moreover, it is a key component for LexA self-cleavage [27]. These processes require the interaction of RecA with single-stranded (ss)-DNA forming a helical nucleoprotein filament [27].

In this paper, we describe a previously unsuspected role for RecA in swarming, but not swimming motility in *E. coli*. The RecA control of swarming motility represents a novel basic RecA role under proliferating conditions beyond its pivotal activities in situations that lead to DNA damage. Our results indicate that this RecA effect on collective swarming migration is possibly mediated through an operative RecA mode requiring neither SOS-induction nor extensive canonical RecA-ssDNA nucleoprotein filament formation.

## Results

### RecA is required for *E. coli *swarming but not swimming motility

**Figure 1:**
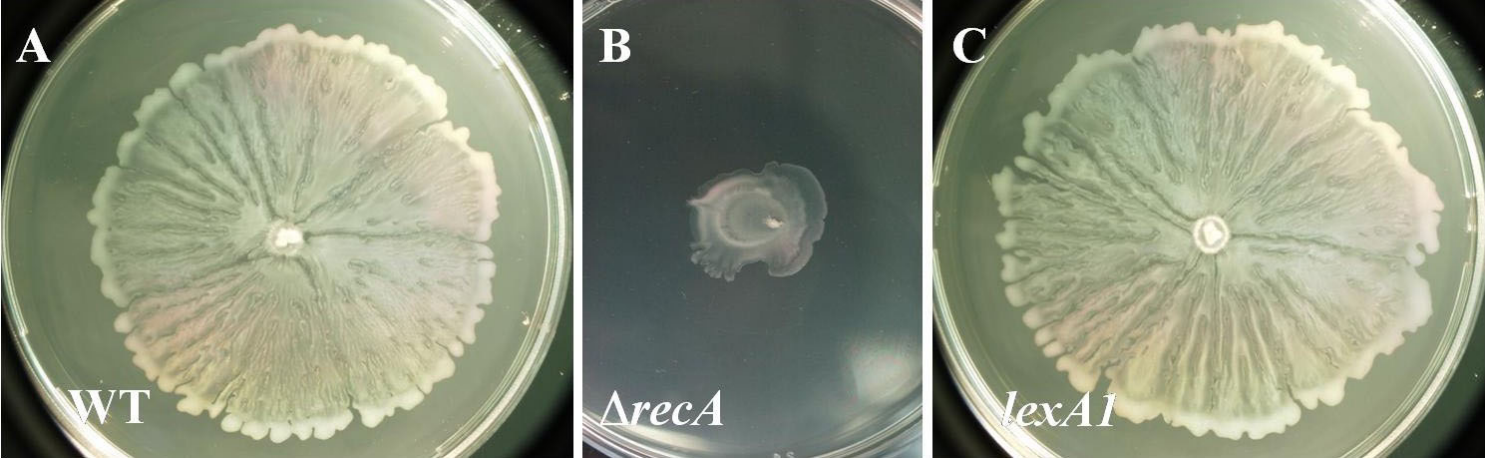
**Swarming colonial patterns**. Swarming colonial patterns developed on a semisolid "Difco" agar surface for 24 h at 37°C by (A) MG1655 wild-type, (B) MG1655Δ(*srlR-recA*)*306*::Tn*10*, and (C) MG1655 *lexA1*. Plates have a diameter of 9 cm.

**Figure 2:**
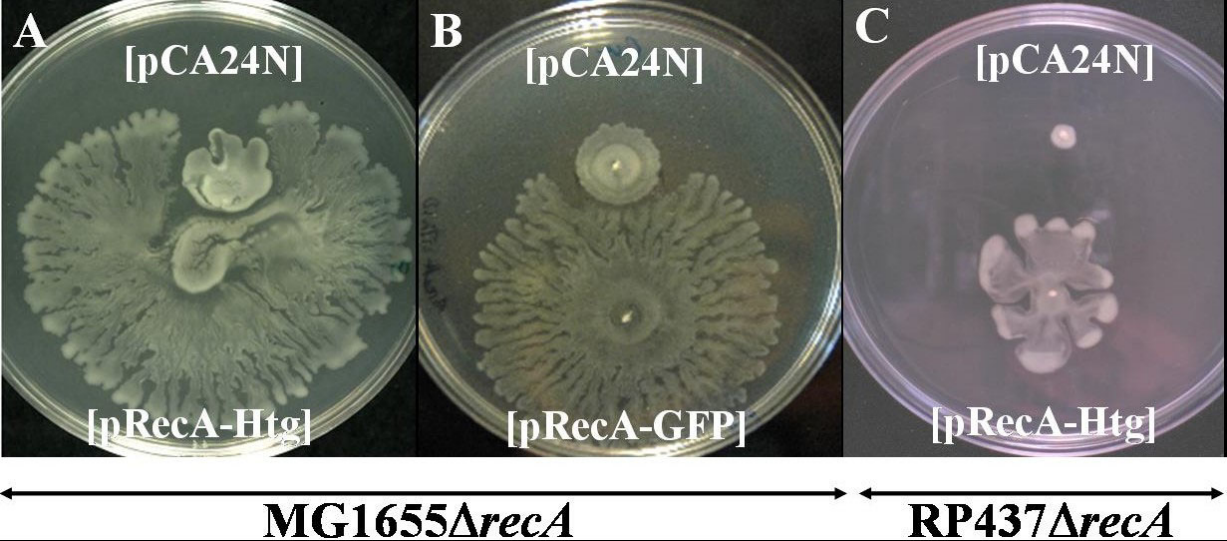
**Swarming complementation test**. (A) *E. coli *JMG1655 Δ*recA *(pCA24N) and JMG1655 Δ*recA *(pRecA-Htg) strains. (B) *E. coli *JMG1655Δ*recA *(pCA24N) and JMG1655Δ*recA *(pRecA::GFP) strains. (C) *E. coli *RP437Δ*recA *(pCA24N) and RP437 Δ*recA *(pRecA-Htg) strains. Plates were photographed after 24 h of development at 37°C. Plates have a diameter of 9 cm.

*E. coli *K-12 MG1655 strain growing on LB-glucose harnessed with 0.5% Difco agar exhibits a vigorous flagella-driven swarming migration, developing a robust colonial pattern associated to this type of motile collective behavior [7] (Figure 1A). While performing experiments designed to study the response of *E. coli *MG1655 to DNA-damage stress, we unexpectedly observed that *E. coli *MG1655 deleted for the *recA *gene was impaired in its ability for swarming migration. This prompted us to study other *recA*-defective strains and we observed that all of them showed identical behaviour. The isogenic MG1655 strains lacking the *recA *gene, JMG1655 Δ(*srlR-recA*)*306*::Tn*10 *(here, Δ*recA306*) or JMG518 Δ*recA-518::kan *(here, Δ*recA518*), were severely impaired in their ability to migrate over semisolid agar surfaces, with a defective outward colonial expansion. Figure 1B shows the swarming colonial pattern of the JMG1655 (Δ*recA306*) strain. To learn whether the absence of RecA was responsible for the defective swarming migration, we carried out complementation experiments. The plasmid pRecA-Htg, harboring an *E. coli recA *wild-type gene that encodes an N-terminal histidine-tagged RecA protein (termed here as RecA-Htg variant), and the empty vector (pCA24N, vector control without *recA*) were introduced by transformation into the wild-type and both Δ*recA *(Δ*recA306 *and Δ*recA518*) strains. Similar results were obtained with MG1655 Δ*recA306 *or Δ*recA518 *derivate strains, hence the results are only shown for one of them: the Δ*recA306 *derivative. Figure 2A shows that the swarming motility of the JMG1655 Δ*recA306 *strain harboring the plasmid pRecA-Htg formed a colonial pattern similar to that of the wild-type MG1655 strain. By contrast, the strain harboring the pCA24N vector was severely impaired in swarming. The ability of the RecA-Htg variant to complement the swarming defect indicates that the lack of RecA is the sole cause of the motility defect in the *recA *mutant strain. This result also demonstrated that the engineered RecA variant (RecA-Htg) protein used in our experiments was capable of promoting the swarming motility process.

**Figure 3:**
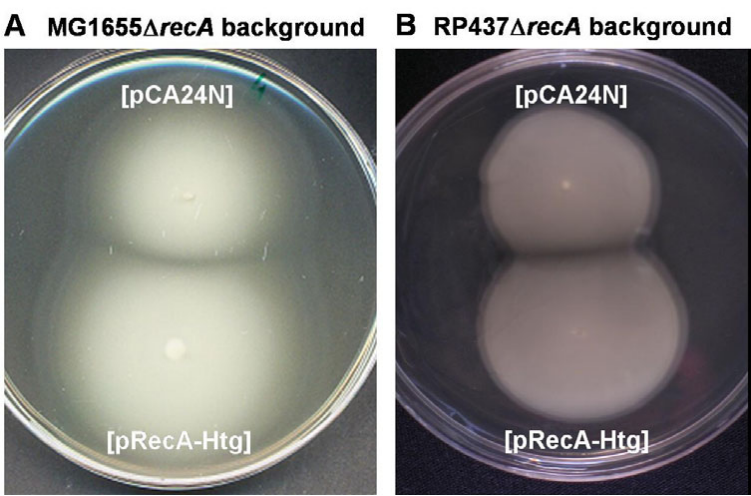
**Swimming motility of *E. coli *MG1655 and RP437 strains**. (A) *E. coli *JMG1655 Δ*recA *(pCA24N) and JMG1655 Δ*recA *(pRecA-Htg) strains. (B) *E. coli *RP437Δ*recA *(pCA24N) and RP437 Δ*recA *(pRecA-Htg) strains. Plates were photographed after 24 h of development at 37°C. Plates have a diameter of 9 cm.

Swimming motility is another well-known *E. coli *flagella-driven motility mode [3,4]. To test whether RecA is also involved in the control of this individual motility process, we examined the swimming ability of the JMG1655 Δ*recA306 *(pCA24N) and JMG1655 Δ*recA306 *(pRecA-Htg) strains. Figure 3A shows that the Δ*recA *mutant only had a slightly reduced swimming colony size. This small difference may be due to the additional time that the Δ*recA *mutant takes at the start of swimming colony expansion, compared to the wild-type strain. However, the speed of the swimming advance was similar in both strains (data not shown). This is consistent with normal flagellation, cellular size, and the individual movement of both swimming cell types (data not shown). Thus, it is likely that RecA is unnecessary for swimming, although it is absolutely essential for swarming motility.

### The RecA effect on swarming motility is not restricted to the *E. coli *MG1655 strain

The ability of *E. coli *strains to swarm on semisolid surfaces was initially described by Harshey and Matsuyama in 1994 [5], and has been studied intensively in the RP437 strain [6]. To know whether the effect of RecA on swarming motility was specific to the strain MG1655, both the swarming and swimming motility behaviour of the RP437 and RP437 Δ*recA *[32] strains were assayed. The swarming capacity of the RP437 Δ*recA *strain was severely impaired when compared to the wild-type RP437 strain. Figure 2C shows that the negative effect produced by the *recA *deletion on the swarming motility of the RP437 strain was almost fully restored by the introduction of the pRecA-Htg plasmid. As in the case of JMG1655 Δ*recA306*, swimming motility was not affected by the Δ*recA *mutation (Figure 3B). These findings indicate that the effect of RecA on swarming motility on "Difco" semisolid surfaces is not restricted to MG1655. Examination of the swarming colonial patterns of JMG1655 (pRecA-Htg) (Figure 2A) and RP437 Δ*recA *(pRecA-Htg) (Figure 2C) also revealed that, under our experimental conditions, swarming of strain RP437 was not as active as that of strain MG1655. In addition, we also observed that when RP437 and MG1655 strains were inoculated together on swarming plates, the MG1655 swarming migration overtook the RP437 swarming ability on semisolid Difco agar surfaces, under the conditions employed in this study (Gómez-Gómez and Blázquez, unpublished results). On the basis of these observations, we decided to use the MG1655 strain as a model for the study of the RecA effect on swarming motility in *E. coli*.

Finally, by a simple visual observation of the colony advance edge of both the wild-type and Δ*recA *strains, we can rule out any effect of RecA on the production of slime because the slime that normally surrounds the colony periphery was, apparently, similar in both cases (data not shown).

### The RecA effect on swarming motility does not require SOS induction

RecA functions as a key component of the regulatory system controlling the induction of the SOS response [27]. The *E.coli *LexA protein represses the transcription of over 40 "SOS response genes", the SOS regulon, including *lexA *itself [33]. The RecA interaction with ss-DNA unveils its coprotease activity (RecA*) that facilitates self-cleavage of the LexA repressor, with a subsequent deregulation of the SOS response genes [27] in an exquisite proportionally-tuned response adjusted to the level of DNA damage [34].

To address whether one or more components of the SOS system could be the direct effector(s) promoting swarming motility, comparative transcriptome expressions between the *recA+ *and Δ*recA *cells, sampled from the edge of colonies grown on swarming plates, were performed by DNA microarray analysis. The obtained results (see Additional file 4) indicated that the transcriptional profile of genes belonging to the SOS regulon [33] were identical in both cases. In addition, an MG1655 derivative, harboring the chromosomal *lexA1 *[Ind-] allele, encoding a well characterized LexA protein resistant to the RecA stimulated LexA self-proteolysis, thus preventing the induction of the SOS response [33], was found to be fully proficient in swarming motility (Figure 1C). Thus, it is likely that the ability of *E. coli *to perform swarming migration on semi-solid surfaces does not require the induction of the SOS response.

### The Δ*recA *strain grown on swarming agar does synthesize flagella yet shows a non-motile colony leading edge without any swarming motion

**Figure 4:**
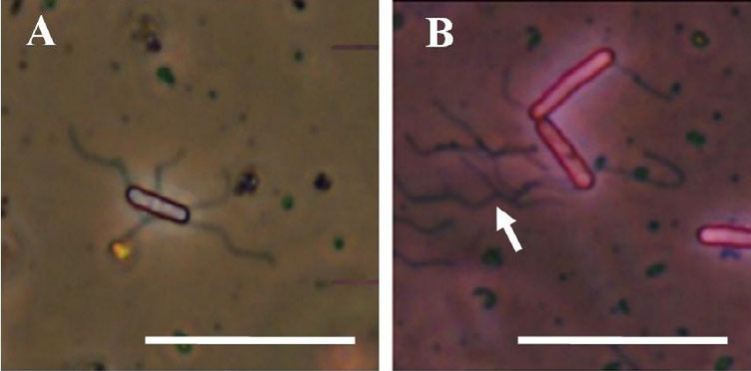
**Flagella filaments**. Visualization of cell flagella extracted from the leading colony edges grown on swarming plates. (A) Typical swarming cell from the JMG1655 Δ*recA *(pRecA-Htg) strain. (B) Typical swarming cells from the JMG1655 Δ*recA *(pCA24N) strain. The arrow indicates the position of the flagella in the preparation. Bar 10 μm.

Optical microscopy was used to determine whether Δ*recA *cells showed defective individual and/or collective movements. Wild-type cells extracted from the swarming colony edge showed a vigorous and rapid individual movement when suspended in an LB liquid drop. However, Δ*recA *cells showed a very slow movement. Direct observation of the leading edge of the swarming colonies indicated that the wild-type colony edge exhibited a vigorous and very rapid convective and collective cell movement, with swirling patterns [7]. In contrast, the leading edge of the Δ*recA *swarming colonies presented a completely resting edge colony (see Additional files 1 and 3). This deficient collective movement of the Δ*recA *strain, leading to a defective colony pattern development, could possibly be due to the absence or damage of the flagella. To test this hypothesis, flagella were stained in both the wild-type and Δ*recA *cells. A total of 200 cells from each strain (10 to 20 different fields) were visualized (see Methods). Figure 4A shows a typical wild-type swarming cell with five or six peritrichous flagella. A total of 159 (79.5%) MG1655 *recA*+ cells and 150 (75%) MG1655 Δ*recA *(pRecA-Htg) cells were similar to the one shown in the figure. The Δ*recA *preparations contained abundant flagella, yet appeared disconnected from the cell (Figure 4B), thereby indicating that Δ*recA *cells have the ability to synthesize flagella. More than 99% (199 out of 200) of the MG1655 Δ*recA *(pCA24N) cells were similar to those shown in the figure.

This result is additionally supported by a comparative DNA microarray analysis between the *recA*+ and Δ*recA *cells grown on semisolid surfaces. This analysis showed that both chemotaxis and flagellar genes related with *E. coli *motility [35] had similar expression levels in both genetic backgrounds (see Additional file 4). Thus, it is likely that the impaired production of flagella is not responsible for the lack of swarming motility of Δ*recA *cells. All together these results suggest that the non-motile behavior of the individual Δ*recA *cells may be due to the impaired flagellar propulsion function necessary to drive the swarming migration process.

### RecA-GFP complementation assays under different proliferating conditions: segregation of RecA functions

**Figure 5:**
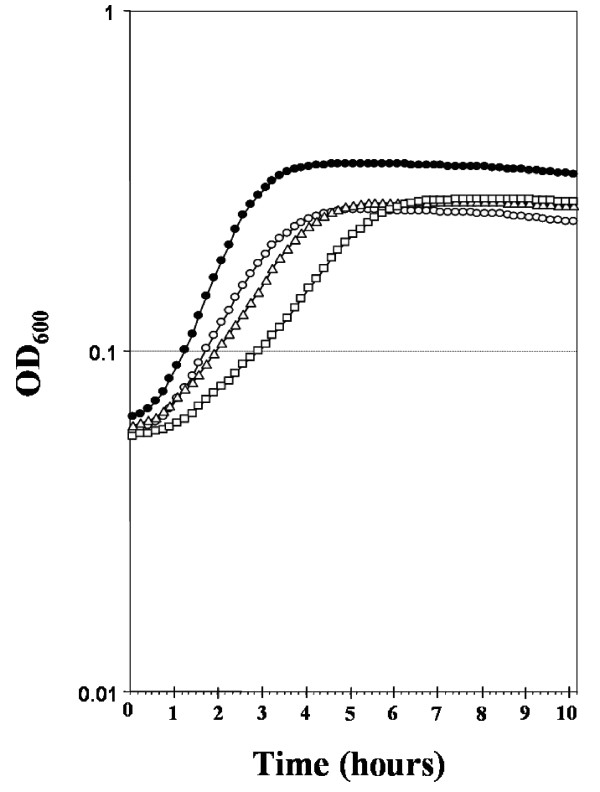
**Growth rates of different strains**. Overnight cultures of *E. coli *strains grown in LB medium were diluted (1/200) and subcultured in fresh LB medium supplemented with 0.5% D-(+)-glucose and chloramphenicol. MG1655 (pCA24N) (filled circles), JMG1655 Δ*recA *(pCA24N) (open circles), JMG1655 Δ*recA *(pRecA-Htg) (open triangles), and JMG1655 Δ*recA *(pRecA-GFP) (open squares)

**Figure 6:**
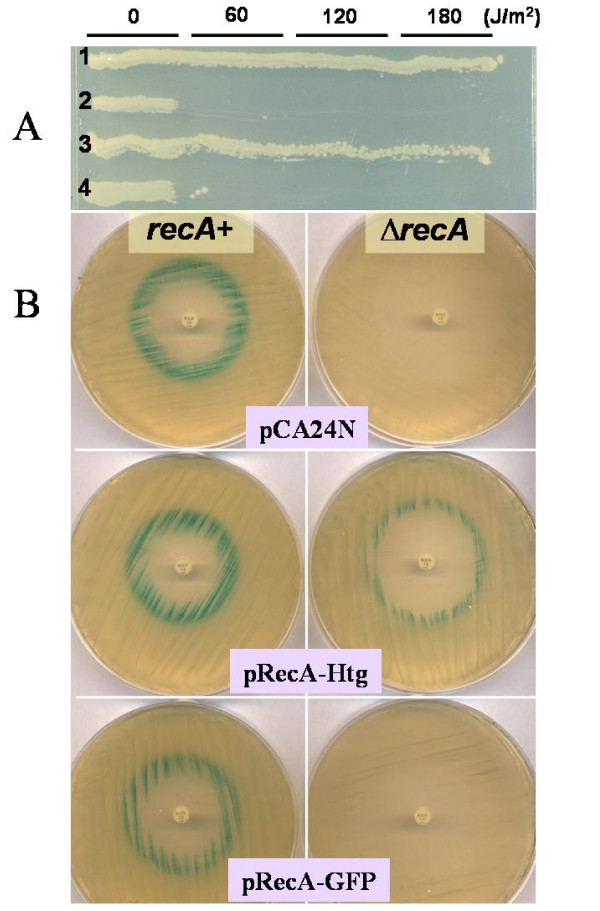
**UV survival and SOS induction assays**. Plasmids pCA24N, pRecA-Htg, and pRecA-GFP were introduced in the strain GW1030 (*dinB*::*lacZ*) [40] and its derivative Δ*recA *[39] by transformation. Norfloxacin-mediated SOS induction of the *dinB*::*lacZ *was assayed by the disk-plate method as described [39]. (A) Survival following UV irradiation of wild-type MG1655 *recA*+ (pCA24N) (1), JMG1655 Δ*recA *(pCA24N) (2), JMG1655 Δ*recA *(pRecA-Htg) (3) and JMG1655 Δ*recA *(pRecA-GFP) (4) strains. UV doses in J/m^2 ^are indicated. (B) The effect of RecA-Htg and RecA-GFP proteins on the norfloxacin-mediated SOS induction of a *dinB*::*lacZ *gene fusion and on sensitivity to norfloxacin. Plates on the left side show the effect of norfloxacin on the wild-type strain GW1030 containing either pCA24N, pRecA-Htg or pRecA-GFP plasmids. Plates on the right side show the effect of norfloxacin on the GW1030Δ*recA *strain containing the indicated plasmids. The intensity of the gray border around the inhibition halo reflects the level of *dinB *transcription [39].

RecA is a multifunctional protein that is involved in DNA recombination, DNA double-strand break repair, induction of the SOS response, SOS mutagenesis and the repair of stalled replication forks [27]. These are, in all cases, functions that depend on RecA-ssDNA nucleofilament formation [27]. To gain further insight into the involvement of RecA in swarming motility, we took advantage of the construction of a RecA-green fluorescent protein fusion (RecA-GFP) [36]. Our reasoning was that this fusion protein could lose its ability to generate the RecA filament formation because of the bulky GFP portion. The ability of the RecA-GFP chimera to complement the defects of the Δ*recA *strain in growth, viability, SOS induction, UV sensibility, genetic recombination and swarming motility was compared to that of the RecA-Htg variant. No significant differences between MG1655 *recA*+ harboring pRecA-Htg, pRecA-GFP or pCA24N (empty vector) in growth, UV survival, viability and genetic recombination were observed. Thus, for simplicity, the complementation behavior of plasmids pCA24N, pRecA-Htg and pRecA-GFP in the Δ*recA *background refers to the strain MG1655 *recA*+ (pCA24N) as a wild-type control. Table 1 and Figures 2A, 2B, 5 and 6 show the results of these rescue experiments with both protein variants and several important results can be extracted from them.

### Swarming motility

Figure 2B shows that the RecA-GFP variant was able to complement the swarming motility defect of Δ*recA *cells to a level similar to the one obtained with the RecA-Htg variant. Direct observation of the swarming colonies' leading edge (see Additional file 2) additionally demonstrated that this variant restored the collective movement completely to a level similar to the wild-type strain (see Additional file 1). Several observations rule out any effect of the GFP moiety of the RecA-GFP protein fusion in swarming motility. Firstly, the pCA24N plasmid, employed as a control vector in our experiments, encodes the GFP protein; however it did not complement the swarming defect of the *recA *mutants (Figure 2A and 2B). Secondly, another different RecA-GFP protein fusion construction encoded in the chromosome of the *E. coli *SS3041 strain [37], which retains an important residual activity promoting UV survival, genetic recombination, and SOS induction [37], showed defective swarming, although normal swimming motility was observed under our conditions (data not shown). Thus, although the biochemical activities associated to the RecA-GFP protein constructed by Renzette *et al *[37] remain to be studied, it seems that: (1) the GFP moiety of the RecA-GFP protein is not involved in the rescue of the swarming defect, and (2) the plasmid-borne RecA-GFP employed in our study [36] and the RecA-GFP protein fusion harbored in the *E. coli *SS3041 strain [37] were different with respect to its effect on swarming motility, suggesting a structural difference between both fusion proteins. However, the possibility that the differential behaviour in swarming motility is due to a gene dosage effect cannot be ruled out.

### Genetic recombination

RecA is essential for genetic recombination [22,28]. As shown in Table 1, RecA-Htg completely restored the production of recombinant Δ*recA *transconjugants to a level similar to the wild-type strain. By contrast, the RecA-GFP protein was remarkably inefficient in carrying out this activity. These results strongly suggest that the RecA-Htg variant was proficient for both DNA pairing and strand exchange between homologous DNA molecules, whereas the RecA-GFP variant was not.

### Growth and swarm cell viability

It has previously been described that an MG1655 derivative Δ*recA *strain shows defective growth [38]. To rule out the possibility that the defective swarming motility of the *recA *mutant was associated to defective growth or decreased viability, both the growth rate and viability of the wild-type and Δ*recA *strains were measured (Figure 5). The growth rate of the Δ*recA *(pCA24N) strain in LB broth supplemented with 0.5% D-(+)-glucose was reduced when compared to the wild-type (harboring the pCA24N plasmid) strain. The Δ*recA *(pRecA-GFP) strain also showed slow growth. The slow growth of the Δ*recA *strain could only be partially restored by the RecA-Htg variant protein. Indeed, even though the RecA-GFP protein appears to exacerbate the slow growth of the Δ*recA *strain, it is still proficient in rescuing its swarming motility defect. These results therefore strongly suggest that the slow growth rate of the Δ*recA *strain is not the cause of the defective swarming motility.

Alternatively, the impaired swarming motility of the Δ*recA *cells may be the result of cell viability loss [25]. This possibility was tested by assessing membrane integrity by staining both the wild-type and Δ*recA *mutant cells isolated from the leading edge of swarming plates with the Live/Dead BacLight Kit (Molecular Probes). A total of 200 cells were visualized in each case. A similar number of cells (196 wild-type and 194 Δ*recA*) appeared viable. This result suggested that the loss of viability was not involved in defective swarming migration.

### UV survival

RecA is required for the recovery of UV-treated cells [25]. As shown in figure 6A lane 3, the RecA-Htg variant was proficient in rescuing the UV-sensitivity phenotype of the Δ*recA *strain, however RecA-GFP was not (Figure 6A, lane 4), suggesting that RecA-GFP is impaired in recovering from the UV-induced DNA lesions, i.e. rescue of the replication fork [25,29] and the recombinational DNA repair [23] both of which required DNA strand exchange.

### SOS induction

Treatment of cells with some agents, such as fluoroquinolone antibiotics, induces SOS response [38,39]. Here we employed the transcriptional *dinB*::*lacZ *gene fusion as a reporter of SOS induction [39,40]. As shown in figure 6B, the RecA-Htg protein supported the norfloxacin-mediated SOS induction, detected as a strong blue/green band around the norfloxacin inhibition halo in a lawn of the Δ*recA *(pRecA-Htg) strain. However, the RecA-GFP protein was unable to complement the defect in SOS induction. By contrast, the RecA-GFP protein was fully inactive in rescuing the Δ*recA *antibiotic sensitivity phenotype, as the size of the norfloxacin inhibition halo for the GW1030 Δ*recA *(pRecA-GFP) strain (4.0 ± 0.2 cm) was as large as that corresponding to GW1030 Δ*recA *(pCA24N) (Figure 6B).

Thus, by using the RecA-GFP fusion protein we have been able to segregate the RecA function on swarming promotion from the other RecA functions. The combination of all our results strongly supports the notion that, although the RecA-GFP protein is proficient in promoting swarming motility, it has lost its capacity to promote SOS induction, recombinational DNA repair and genetic recombination.

### Biochemical studies on ssDNA-dependent ATPase activity of the RecA-GFP protein fusion

**Figure 7:**
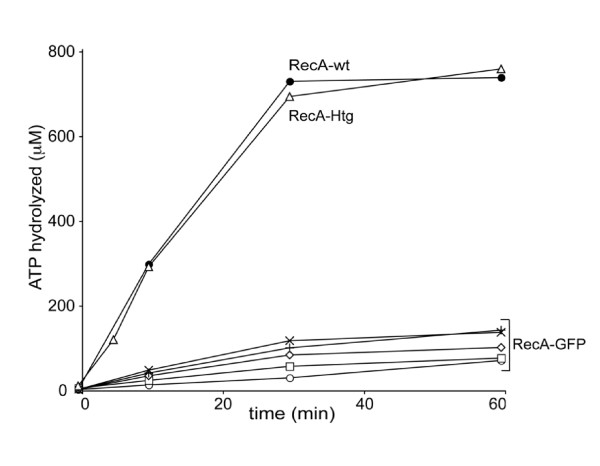
**RecA or RecA-GFP catalyzed ssDNA-dependent ATP hydrolysis**. The RecA protein [1 μM (filled circles)] or the RecA-Htg variant [1 μM (triangles)] or increasing concentrations of RecA-GFP [0.25 (circles), 0.5 (squares), 1 (rhombuses), 1.5 (plus signs) or 2 μM (crosses)] were preincubated with circular ssDNA (10 μM) in buffer C, then 2 mM ATP was added and the reaction was incubated for a variable time at 37°C. Time zero corresponds to the moment the ATP was added.

The above results obtained with the RecA-GFP protein fusion suggest the possibility that RecA promotes swarming motility via a molecular mode, independently of the RecA filament formation and/or DNA strand exchange. The RecA-Htg and RecA-GFP proteins were purified and biochemically characterized. ATP binding induces RecA assembly on ssDNA and ADP induces RecA disassembly from DNA [24]. Therefore, ATP hydrolysis is an indirect measure of RecA protein binding to ssDNA, and under most condition the rate of ATP hydrolysis correlates to the amount of RecA bound to ssDNA. The RecA, RecA-Htg or RecA-GFP catalyzed ssDNA-dependent ATP hydrolysis was assayed using half-saturating protein concentrations (1 monomer/10 nucleotides) in buffer C containing 20 mM NaCl and 2 mM ATP (see Methods). The rate of RecA and RecA-Htg-mediated ATP hydrolysis increased during the first 30 min after binding to ssDNA, reaching levels similar to those observed in the wild-type RecA protein [28] (Figure 7). In the absence of ssDNA, ATP hydrolysis by wild-type RecA, RecA-Htg or RecA-GFP was just above the background (data not shown).

The ATPase activity of the RecA-GFP variant was 8 to 10-fold lower when compared to the RecA-Htg variant (Figure 7) or wild-type RecA protein [24,28] (Figure 7). The presence of increasing concentrations of RecA-GFP slightly enhanced ATPase activity (Figure 7). A modification of the pH in the reaction mixture exerted either a negative effect (1.8-fold reduction) at pH 8.0 or a positive effect (1.5-fold increase) at pH 6.0 where RecA binds double stranded (ds) DNA [24,28] (data not shown). The ATPase activity of RecA-Htg or RecA-GFP variants was strongly inhibited by prebound SSB to ssDNA (data not shown). Therefore, it is improbable that we are measuring the activity of a possibly contaminating ssDNA-dependent ATPase.

**Figure 8:**
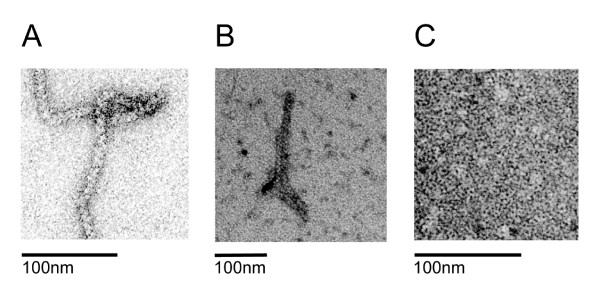
**Visualization of filament formation of RecA or its variants**. Circular ssDNA (10 mM in nucleotides) was pre-incubated with (1 mM) RecA (A) or variants RecA-Htg (B) or RecA-GFP (C) 20 min at 37°C in buffer C containing 20 mM NaCl and 1 mM ATPγS, and incubated for 40 min at 37°C. The specimens were prepared for electron microscopy by negative staining with 1%

If long nucleoprotein filaments are to be formed, the inherent rate of extension of the RecA filaments during assembly should be faster than the end-dependent disassembly. We have shown that RecA-GFP catalyzes ATP hydrolysis less efficiently than RecA-Htg or wild-type RecA protein and assumed that RecA-GFP might promote end-dependent disassembly at a very low rate and perhaps, under these conditions, long nucleoprotein filaments could be formed. To address this hypothesis, the wild-type RecA or the variants RecA-Htg or RecA-GFP (1 μM) were incubated with circular ssDNA in the presence of 1 mM ATPγS and the reaction visualized by electron microscopy. As observed for RecA (Figure 8A) [41], the RecA-Htg-ssDNA complex shows an extended helical conformation in the presence of ATPγS (Figure 8B). Under standard conditions, using 1 μM RecA-Htg, 8–16 nucleoprotein filaments per field were observed (data not shown). However, under identical conditions, we have failed to detect long RecA-GFP nucleoprotein filaments on circular ssDNA in the presence of ATPγS after analysing more than 20 different fields (Figure 8C). Only RecA-GFP blobs were observed even in the presence of 4 μM RecA-GFP (C. M. and R. Lurz, unpublished results). Therefore, we can conclude that quite probably the GFP moiety inhibited the RecA nucleation step in RecA-GFP filament formation.

Under typical assay conditions, ATP acts in combination with RecA or its RecA-Htg variant to promote pairing and strand exchanges [28]. RecA-GFP, however, failed to promote strand exchange when using a circular ssDNA and a *Kpn*I-linearized homologous dsDNA.

Overall these biochemical data are consistent with the results obtained from *in vivo *genetic recombination experiments (Table 1). In combination, the results of genetic complementation and biochemical assays presented in the previous sections strongly support the notion that the RecA-GFP protein, although proficient in promoting swarming motility, does not have the capacity to promote both SOS induction and homologous recombination, activities requiring an extensive canonical RecA-ssDNA filament formation. However, the possibility that a residual localized RecA-GFP short filament unable to reach a sufficient level to induce the SOS could be responsible for this activity in swarming cells cannot be ruled out. A short RecA-GFP filament formation could explain the residual *in vivo *genetic recombination observed in the Δ*recA *(pRecA-GFP) strain (Table 1).

## Discussion

In this paper, we present experimental evidence supporting the fact that the RecA protein, described as the prototypic member of the evolutionarily-conserved RecA/RAD51 family of proteins [42], is required for *E. coli *K-12 swarming but not swimming motility. The RecA protein plays a key role in recombinational DNA repair and genetic recombination processes via its biochemical activity of pairing and exchanging DNA between two homologous chromosomes [22-24,42,43]. By forming an active filament on ssDNA, RecA controls the induction of the SOS response, promotes the generation of the SOS-induced DNA polymerase V (polV) active complex (UmuD'_2_C) [27], and furthermore the RecA filament has the ability to interact with polV in *trans *to stimulate its activity on translesion DNA synthesis (TLS), a SOS mutagenesis pathway, in response to chronic DNA damage [30,31]. The results presented here expand on our knowledge of the physiological processes affected by this important protein and reveal a hitherto unknown role for RecA in the *E. coli *physiology: the promotion of swarming motility. Although it has been described that *Salmonella enterica *Serovar Typhimurium mutant strains with LPS defects are differentially affected in their abilities to swarm and swim [15], RecA is the first reported cellular factor that specifically affects swarming but not swarming motility in *E. coli*.

At least two models could explain the RecA requirement for swarming motility. In the first one, RecA interacts with one or several cellular component(s) expressed (or activated) during the swarming differentiation process, but not during swimming motility. This model implies that RecA could influence the expression of a certain gene(s) in swarming cells, however, the results of our transcriptome analysis indicated that the expression of a hypothetical component(s) in swarming cells seems to be a RecA-independent process, because RecA does not influence gene expression in swarming cells. This is consistent with the observation that RecA-GFP fails to induce the SOS response, although it does promote swarming motility. In the second model, RecA may undergo a "modification" (e.g posttranslational) to reach an activated form that allows swarming motility.

Regardless of the molecular mechanism behind RecA-dependent swarming motility, RecA must interact with an unknown cellular target(s) to promote this motility. Recent global protein interaction network experiments in *E. coli *revealed a direct physical interaction between the RecA and the CheW protein [44]. The CheW protein is a cytoplasmic linker of the chemotactic signal transduction system that couples the membrane chemoreceptors to the intracellular cytoplasmic phosphorylation cascade [4]. This cascade controls the direction of flagellar motor rotation [4], which is an essential component in the mechanical control of the chemotaxis system on swarming motility in *Salmonella enterica *serovar Typhimutium [45]. Thus, it is tempting to speculate that a RecA-CheW physical interaction, active in swarming but not in swimming cells, could be the direct link between RecA and swarming motility.

All functions so far described for the RecA protein are related to their fundamental biological role in the maintenance of genome integrity and variability. These functions require the formation of an extensive RecA-ssDNA nucleofilament complex as a central molecular scaffold that drives the RecA activities in homologous recombination, SOS induction and SOS mutagenesis. The RecA-ssDNA nucleofilament formation is a very orchestrated, dynamic and precise molecular process during DNA strand exchange [27,46]. In addition, the cells have several molecular modulators, such as the SSB, single-stranded DNA-binding protein, RecFOR proteins, DinI, RecX and RdgC influencing RecA's filament extension [27,47,48]. It is widely accepted that under most conditions the rate of ATP hydrolysis by the RecA protein correlates with the amounts of RecA bound to ssDNA [22,24]. Although the RecA-GFP protein used in our studies retained a residual ssDNA-dependent ATPase activity, it completely lost its DNA strand exchange ability, a process requiring an extensive RecA-ssDNA nucleofilament formation. Despite this impaired *in vitro *biochemical activity, RecA-GFP is proficient in complementing the defective swarming motility of a Δ*recA *mutant. We consider that the segregation of the RecA effect on swarming motility from those that require the formation of long canonical RecA-ssDNA (or RecA-dsDNA) nucleoprotein filaments, favor the hypothesis that the formation of these long filaments is not strictly necessary for swarming motility. Alternative models, including a RecA activity independent of any DNA interaction cannot, however, be discarded.

The integration of two, apparently disconnected, biological processes, such as the maintenance of genome integrity and motility in a unique protein, may have important evolutive consequences. Adaptive biological evolution, relying upon the existence of genetic variability in a population of organisms generated by mutation and recombination and upon the natural selection of genotypes with better fitness, also depends on the capacity of the fittest to colonize the niche. The existence of a molecule able to receive signals informing on the status of genome stability and transmitting them to cellular systems, which in turn permit rapid spatial migration, could be an important factor for improving the colonization of a new ecological niche. Thus, it is tempting to speculate that the RecA protein could be the molecule that links those apparently independent molecular processes, permitting bacteria to manage the different cellular processes in a coordinated manner, influencing their adaptive capacity.

An important goal in future research will be to determine the ultimate molecular entity and the operative molecular mechanism through which RecA allows swarming motility in *E. coli *to proceed. The possibility of this novel function being conserved in other RecA homologues should also be explored.

## Conclusion

A novel physiological role for *E. coli *RecA, a protein previously known as a key multifunctional cellular factor required for the maintenance of genome integrity under stress, has been described in this work. RecA participates actively in the promotion of *E. coli *swarming motility but not swimming motility. While the ultimate operative molecular mode through which RecA acts to control *E. coli *swarming motility is waiting to be disclosed, the RecA effect on swarming motility appears to be independent of an extensive canonical RecA nucleofilament formation. The integration of two apparently disconnected biologically important processes, such as the maintenance of genome integrity and motility in a unique protein, may have important evolutive consequences.

## Methods

### *E. coli *strains, plasmids and growth conditions

The *E*. *coli *wild-type strain used in this work was MG1655 (CGSC6300) obtained from the *E. coli *Genetic Stock Center [7]. The RP437 and RP437 Δ(*recA*)*Sst*II-*Eco*RI *srl*::Tn*10 *strains were a gift from R.B. Bourret. The MG1655 strain harbouring the *lexA1 *[Ind^-^] allele was obtained from Ivan Matic (Necker hospital, Paris). All strains were routinely grown in Luria-Bertani (LB) medium (1.0% "Difco" Bacto-tryptone and 0.5% "Difco" Yeast Extract, 0.5% NaCl) or on LB plates. When necessary, media were supplemented with the following antibiotics used at the indicated concentrations: chloramphenicol (Cm) 20 μg/ml; tetracycline (Tc) 20 μg/ml and kanamycin (Km) 30 μg/ml. The MG1655Δ*recA *derivatives, JMG1655 and JMG518, were constructed by P1vir-mediated transduction [49] of the Δ*recA *alleles from the SMR624 Δ(*srlR-recA*)*306*::Tn*10 *[50] and MS6 Δ*recA-518::*kan [51] strains respectively. ELE1 is the *E.coli *K-12 Hfr strain P4X [52] with the Δ*fhuD*::kan marker, transferred by P1vir transduction from the JW0184 strain obtained from the *E*. *coli *K-12 Genobase library Keio collection of NARA Institute [53]. The *recA *and *lexA1 *phenotypes were checked by UV sensitivity. Induction of the SOS system was assessed with strains GW1030 [40] and GW1030 Δ(*srlR-recA*)*306*::Tn*10 *as described [39]. All plasmids were obtained from the ASKA library of the NARA Institute [36]. The plasmid pRecA-Htg harbors an *E. coli recA *wild-type gene encoding an N-terminal histidine-tagged RecA protein (termed here as RecA-Htg variant), whereas the pRecA-GFP plasmid harbors this modified *recA *gene fused with the *gfp *gene (green fluorescent protein) that renders a RecA-GFP protein fusion. Expression of the genes encoding the RecA-Htg or RecA-GFP proteins is under the control of an IPTG- (isopropyl beta-D-thiogalactoside)-inducible promoter, which is strictly repressed by the LacI repressor expressed from the PlacI^q ^promoter. Plasmid pCA24N was the cloning vector, encoding a non-fused GFP protein. A detailed construction of those plasmids has been described previously [36].

### Conjugation experiments

Matings were performed in LB at 37°C for 1 h with donor ELE1 (*Hfr ΔfhuD::*kan) and the receptor strains harboring pCA24N (Cm^R^) plasmid or its derivatives. Transconjugants were selected on LB plates supplemented with chloramphenicol and kanamycin.

### Determination of growth curves

Strains were cultured overnight in LB. A 1/200 dilution was carried out in fresh LB and incubated in 96-well plates. The plates were incubated at 37°C with agitation in an Infinite M200 multiwell fluorimeter (Tecan, Switzerland). Optical density at 600 nm was recorded every 10 min for 10 h. Each point of the curve represents the average value of four measurements in four different parallel experiments.

### Viability assays

Wild-type and Δ*recA *cells were isolated from swarming plates and stained with the Live/Dead BacLight Kit according to the manufacturer's instructions (Molecular Probes, Eugene, Oreg).

### Motility assays

Overnight cultures of the different strains were grown in LB and inoculated with a sterile toothpick on swimming and swarming plates. The swimming motility plates were prepared with 0.3% "Difco" agar, 1.0% "Difco" Bacto-tryptone, 0.5% "Difco" Yeast Extract and 0.5% NaCl [7]. The swarming motility plates were prepared with 0.5% "Difco" agar, 1.0% "Difco" Bacto-tryptone, 0.5% "Difco" Yeast Extract, 0.5% NaCl and 0.5% D-(+)-glucose. The plates were dried for 5–6 h at room temperature before being inoculated and were photographed after a 24-h incubation at 37°C. When the assays were performed with strains carrying pCA24N or its derivatives, the plates contained chloramphenicol.

### Flagella visualization

Cells isolated from the periphery of a swarming colony were deposited onto a microscope slide with a 10 μl LB drop and covered with a coverslip. Flagella staining was carried out as described [54]. The slide was propped vertically and 10 μl of dye was applied to the top edge of the coverslip to stain the sample by capillary action. A total of 200 well-stained cells from each strain were observed by means of an Olympus BX61 microscope under 1,000 × magnification. Images were recorded with the image capture software of the DP70 CCD camera coupled to the microscope.

### Transcriptome analysis of swarm cells

The cells from the periphery of six wild-type and Δ*recA *colonies grown on swarming plates were collected, resuspended in saline solution, and harvested by centrifugation. Total-RNA was isolated with the RNeasy mini kit (QIAGEN, Valencia, CA) and Dnase-I digestion was performed on-column. RNA samples were quantified, checked by gel electrophoresis and stored at -80°C until further use. RNA preparations from each sample were used as templates for cDNA synthesis by employing 10 μg of total RNA as template for reverse transcriptase to produce cDNA labeled with either Cy3- or Cy5-dCTP (Superscript™ Indirect cDNA labeling System). Two different array hybridizations were performed using RNA samples extracted from six independent swarming colonies for the wild-type and the Δ*recA *under analogous conditions. To correct for possible differences in Cy3 and Cy5 dye incorporation, the cDNAs were labeled with Cy3 dye in two hybridizations and with Cy5 dye in the other two (dye swapping). Thus, four independent array hybridizations were performed and analyzed as described.

The *E. coli *DNA microarrays, containing more than 95% of the 4,290 ORFs identified in the *E. coli *K12 genome, were purchased at the Wisconsin Gene Expression Center, (University of Wisconsin). Prehybridization was performed at 42°C for 30–45 min in 6 × SSC, 0.5% SDS and 1% BSA, and slides were rinsed five times with distilled water. Cy5 and Cy3 cDNA probes were mixed (300 pmol of each label) in a final volume of 70 μl of hybridization buffer (50% formamide, 3 × SSC, 1% SDS, 5 × Denhardt's and 5% Dextransulphate). The probe was denatured at 95°C for 5 min and applied to the slide using a LifterSlip (Erie Scientific, Portsmouth, NH). Slides were then incubated at 42°C for 16 h in hybridization chambers (Array-It) and subsequently washed sequentially in the following solutions: twice in 0.5 × SSPE, 0.1% TWEEN20, twice in 0.5 × SSPE and finally in 0.1 × SSPE for 5 min each. Slides were dried by centrifugation at 563 g for 1 min before scanning. Images from Cy3 and Cy5 channels were equilibrated and captured with a GenePix 4000B (Axon, Molecular Devices, Union City, CA) and spots quantified using GenPix software (Axon). The data from each scanned slide were first escalated and normalized using the Lowess method, and then log-transformed. The mean of the log-ratio intensities and the standard deviation among replicates were generated. Two statistical approaches were used to identify differentially regulated genes: a t-test and a z-score. A strong stringency was used. Only genes with a gene signal >50, p-value < 0.05, Z-score >2.5 or < 3.0 and fold change >3 or < -3 were considered differentially expressed.

### Survival following UV irradiation

A fresh overnight culture was evenly applied with a loop on the surface of a square 12 cm plate containing Luria-Bertani medium harnessed with agar (15 g/L) supplemented with chloramphenicol and 0.5% D-(+)-glucose. The plate was partially covered by a sheet of paper foil and placed under a UV (ultraviolet light) lamp (254 nm). The foil was progressively retracted following three exposures of 60 J/m^2^. The irradiated plate was incubated in the dark at 37°C for 24 h.

### SOS induction assays

SOS induction was assessed by measuring the norfloxacin-mediated SOS induction of a transcriptional *dinB::lacZ *gene fusion, as previously described [39].

### Time-lapse videos

The leading edges of the JMG1655Δ*recA *(pCA24N); JMG1655Δ*recA *(pRecA-Htg) and JMG1655Δ*recA *(pRecA-GFP) colonies grown on swarming plates were captured. Magnification, 1,000 ×; frame capture rate, 3 frames/s taken with a CCD DP70 camera coupled to a BX61 Olympus microscope.

### Protein purification

*E. coli *MG1655 cells bearing either pRecA-Htg or pRecA-GFP plasmids were used to overexpress the RecA-Htg and RecA-GFP proteins, respectively. *E. coli *MG1655 cells bearing the pRecA-Htg plasmid were grown at 37°C to middle exponential phase and the expression of the RecA-Htg protein was induced for 120 min by adding IPTG. Cells were harvested, resuspended in buffer A (50 mM potassium phosphate pH 7.5, 10 mM β-mercaptoehanol, 10% glycerol) containing 50 mM NaCl, disrupted by sonication and centrifuged. The RecA protein supernatant was loaded onto a Q-sepharose column (Pharmacia, Sweden) equilibrated with the same buffer. The flow-through was then loaded onto a Niquel column (Qiagen, Valencia, CA) equilibrated with buffer A containing 50 mM NaCl. The Niquel column was washed with a 10-column volume of buffer A containing 1 M NaCl and 20 to 90 mM imidazol. The RecA-Htg protein was eluted with buffer A containing 500 mM NaCl and a 100 to 200 mM imidazol gradient. Fractions containing RecA-Htg protein were recovered and loaded onto a Hydroxyapatite (HA) column (BioRad, Hercules, CA) equilibrated with the same buffer containing 200 mM NaCl. The column was washed with buffer A and eluted with a linear 100 to 200 mM K^+ ^phosphate gradient containing 200 mM NaCl. Fractions containing RecA-Htg were recovered and dialyzed against buffer B (50 mM Tris-HCl pH7.5, 50% glycerol) containing 200 mM NaCl and stored at -20°C. *E. coli *MG1655 cells bearing pRecA-GFP plasmid were grown to middle exponential phase at 30°C and expression of the RecA-GFP protein was induced for 200 min by adding IPTG. Cells were harvested, resuspended in buffer A (50 mM K^+ ^phosphate pH 7.5, 10 mM β-mercaptoethanol, 10% glycerol) containing 1 M NaCl, disrupted by sonication and centrifuged. The fraction of the RecA-GFP protein found in the supernatant was loaded onto a Niquel column equilibrated with the same buffer containing 1 M NaCl. The Niquel column was washed with a 10-column volume of buffer A containing 1 M NaCl and 20 mM imidazol and eluted with buffer A containing 1 M NaCl and a 50 to 80 mM imidazol gradient. Fractions containing the RecA-GFP protein were dialyzed against buffer B containing 1 M NaCl and loaded onto a HA column equilibrated with the same buffer. The RecA-GFP was eluted with buffer B containing 50 mM potassium phosphate and 1 M NaCl. Fractions containing RecA-GFP were recovered and dialyzed against buffer B containing 150 mM NaCl and stored at -20°C. The wild-type RecA protein was purchased from Amersham Biosciences (GE, Sweden). Protein concentration was determined by the Bradford method and expressed as mol of protein monomers.

### ATPase activity measurement

Standard reactions were incubated for an indicated time at 37°C in buffer C (50 mM (Tris-HCl pH7.5, 10 mM magnesium acetate, 5% glycerol) containing 20 mM NaCl with circular 3,199-nucleotide pGEM ss-DNA (10 μM in nucleotides) and the indicated amounts of RecA-Htg or RecA-GFP proteins in a volume of 20 μl. Incubations were performed at 37°C at different time intervals and in 20 μl total volume. Different ATP concentrations were used, containing 20 nM of [γ-^32^P]-ATP (1:100,000) for 15 min at 37°C in buffer C. The ATPase activity was determined by thin-layer chromatography, as previously described [55].

### DNA three-strand exchange reaction

The 3,199-bp *Kpn*I-cleaved pGEM ds-DNA (20 μM in nucleotides), and homologous circular 3,199-nucleotide pGEM ss-DNA (10 μM in nucleotides) were incubated with RecA or RecA-GFP (2 μM) and SSB (1 μM) in buffer C containing 40 mM NaCl and 1 mM ATP 60 min at 37°C. The samples were deproteinized as described [56,57], and fractionated through 0.8% AGE with Ethidium Bromide. The signal was quantified using a Phosphorimager (Amersham Biosciences-GE, Sweden).

### Electron microscopy

Circular pGEM ss-DNA (10 μM) was pre-incubated with various amounts of RecA, RecA-Htg or RecA-GFP for 20 min at 37°C in buffer C containing 20 mM NaCl and 1 mM ATPγS, and incubated for 40 min at 37°C. DNA-protein complexes were visualized by negative staining with 1% uranyl acetate [58].

## Authors' contributions

JMGG performed the first phenotypic observation. JMGG and JB participated in the design of the study and carried out phenotypic and molecular genetic studies. They drafted, articulated and wrote the entire manuscript. Both are considered as corresponding authors. JCA participated in the design of the biochemical studies and drew up the biochemical results. CM carried out the biochemical studies.

## Supplementary Material

Additional file 4 **Transcriptome analysis of swarm cells**. Results from the comparative transcriptome expressions between the *recA+ *and Δ*recA *cells sampled from the edge of colonies grown on swarming plates, in Microsoft Excel spreadsheet format. Click here for file

Additional file 1 **Dynamics of the cellular collective swarming motility of the JMG1655 Δ*recA *(pRecA-Htg) strain**. Time-lapse video in AVI format recording the vigorous collective swarming movement observed in the advanced colonial edge of the JMG1655 Δ*recA *mutant bearing the pRecA-Htg plasmid. Click here for file

Additional file 3 **Inactive cellular swarming motility of the JMG1655Δ*recA *(pCA24N) strain**. Time-lapse video in AVI format showing the inactive collective movement observed in the colonial edge of the JMG1655Δ*recA *(pCA24N) strain. Click here for file

Additional file 2 **Dynamics of the cellular collective swarming motility of the JMG1655 Δ*recA *(pRecA-GFP) strain**. Time-lapse video in AVI format showing the vigorous collective swarming movement observed in the advanced colonial edge of the JMG1655 Δ*recA *mutant bearing the pRecA-GFP plasmid. Click here for file

## Figures and Tables

**Table 1:** The effect of RecA and RecA-GFP on genetic recombination.

**Strain**	***Recombination frequency***	***Relative recombination***
*recA*^+ ^(pCA24N)	2.3 ± 1.1 × 10^-2^	1.0
Δ*recA *(pCA24N)	6.8 ± 2.3 × 10^-7^	2.9 × 10^-5^
Δ*recA *(pRecA-Htg)	8.5 ± 3.2 × 10^-3^	0.36
Δ*recA *(pRecA-GFP)	7.4 ± 4.5 × 10^-6^	3.2 × 10^-4^

Matings were performed as described in Methods. The recombination frequency is the number of transconjugants [Cm^R^, Km^R^] relative to the number of donor cells. Relative recombination indicates recombination values relative to MG1655 *recA*+ (pCA24N). Results represent the average of three independent measurements.
